# Construction of paclitaxel-based antibody–drug conjugates with a PEGylated linker to achieve superior therapeutic index

**DOI:** 10.1038/s41392-020-00247-y

**Published:** 2020-07-29

**Authors:** Ting Shao, Tianzhi Chen, Yuning Chen, Xiaoyue Liu, Yi-Li Chen, Qi Wang, Tong Zhu, Maojun Guo, Hui Li, Dianwen Ju, Chunhe Wang

**Affiliations:** 1 Biotherapeutics Discovery Research Center, Shanghai Institute of Materia Medica, Chinese Academy of Sciences, Shanghai, 200126 China; 2 Dartsbio Pharmaceuticals, 528400 Zhongshan, Guangdong China; 3 Levena Biopharma, B8-301, 218 Xinghu Street, SIP, Suzhou, Jiangsu 215123 China; 4 Department of Biological Medicines & Shanghai Engineering Research Center of Immunotherapeutics, Fudan University School of Pharmacy, 201203 Shanghai, China

**Keywords:** Drug development, Drug development

**Dear Editor,**

The selection of linkers and payloads plays a crucial role in determining the therapeutic indices of antibody–drug conjugates (ADCs).^1^ Valine–citrulline (Val–Cit) coupled with a self-immolative ρ-aminobenzyl (PAB) spacer as a cleavable dipeptide linker, designated as “VC linker” in this letter, has been popularly used in ADC conjugation.^1^ However, VC linker is highly hydrophobic^2^ and, together with hydrophobic payloads at high DARs (drug-to-antibody ratios), may turn ADC molecules that have hydrophilic parts in their antibody portions, into aggregation-prone “surfactant-like” structures. Aggregation of ADCs in vivo may lead to accelerated plasma clearance, suboptimal efficacy, and increased toxicity.^2,3^

Trophoblastic cell-surface antigen-2 (Trop-2) is not only overexpressed in a variety of solid human carcinomas, but also expressed at variable levels in crucial normal tissues like skin and mucosa, making it a druggable but risky target for ADC therapies.^4^ IMMU-132, a Trop-2-targeting ADC molecule using SN38, a moderate-toxicity payload, together with a PEGylated acid-labile linker, has been approved by the FDA in April 2020 for treating triple-negative breast cancer (TNBC). However, another Trop-2-targeting ADC molecule, RN927C, which adopts more toxic auristatin-derived payload Aur0101 and a hydrophobic linker, was terminated early in Phase I clinical trial (NCT02122146) due to excess toxicity, highlighting that the key of success in targeting Trop-2 with ADC technologies is to screen for optimal linker and payload combinations in order to achieve the highest therapeutic index.

As paclitaxel (PTX) has been used therapeutically in pancreatic cancer and TNBC, in which Trop-2 is highly expressed, it appears to be a viable payload option for Trop-2-targeting ADCs. However, poor aqueous solubility of PTX has impeded its therapeutic application in carcinomas.^5^ To overcome solubility problem, more hydrophilic PTX derivatives and prodrugs, as well as ADCs,^5^ have been tested. Up to date, however, PTX-conjugated ADCs failed to exhibit sufficient anticarcinogenic effects in vivo, or did they progress to clinical development stage.^5^ The cause of unsatisfactory preclinical results has not been fully understood, but maybe in part by the simultaneous use of hydrophobic linkers with ultra-hydrophobic PTX.

In a separate study (unpublished), we have optimized PEGylation in MMAE-conjugated ADCs and found that replacing citrulline with lysine in a cleavable dipeptide “VC linker” and incorporating linear PEG24 to lysine’s free amino group as a parallel branch (peg4-Val-Lys(PEG24)-PAB, designated as “VK linker” in this letter, see (Fig. 1a) rendered the most stability and hydrophilicity to ADC molecules. By using the VK linker, we successfully generated a stable PTX-conjugated ADC and, designated as hRS7-VK-PTX (Fig. 1a), with a high DAR value at 8 (Fig. 1b). However, our effort to conjugate PTX to hRS7 through a hydrophobic VC linker failed because this molecule precipitated completely from solution during preparation, supporting the concept that combining hydrophobic linkers with ultra-hydrophobic payloads would impair molecular stability of ADC molecules. For comparison purpose, we also conjugated payload MMAE (auristatin E) and SN38 to hRS7 with DAR value at 8, namely hRS7-VK-MMAE and hRS7-VK-SN38 (Fig. 1b; Supplementary Figs. 1 and 2a). MMAE cannot be used as a free drug therapeutically due to excessive toxicity, but has gained popularity as an ADC payload.^1^ SN38 is a moderate-toxicity payload used in IMMU-132 mentioned above. Both MMAE and SN38 are hydrophobic but not as much as PTX (data not shown). After conjugation to hRS7 with a VK linker, none of the three ADCs showed severe aggregation and degradation even after incubation at 60 °C for 1 h (Supplementary Fig. 2c, d, e), indicating that PEGylation linker can improve the stability of ADC molecules with hydrophobic payloads.

**Fig. 1:**
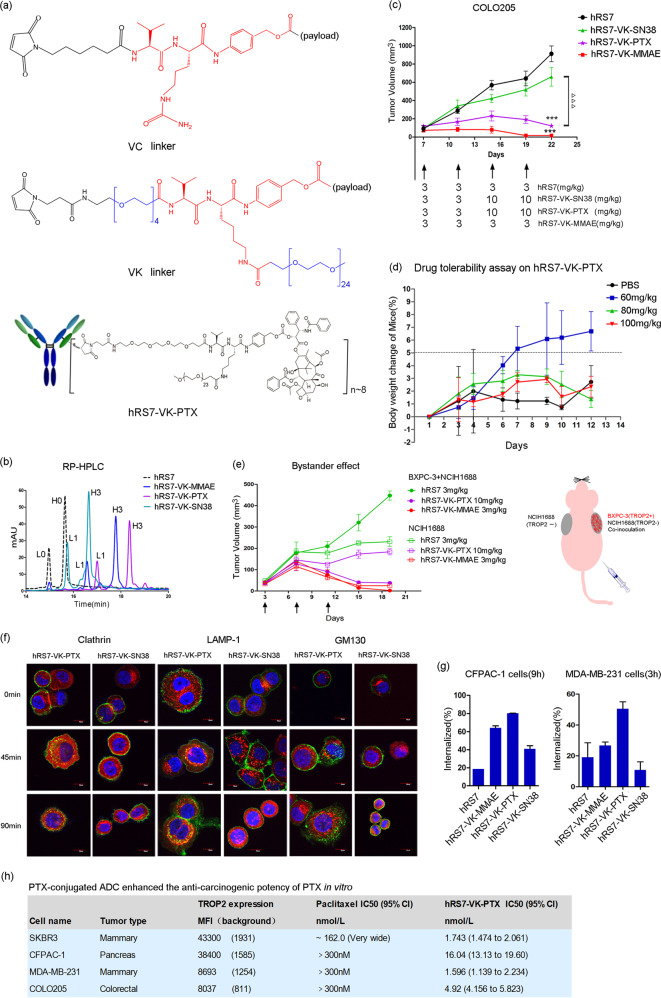
**a** Molecular structures of VC (Val–Cit-PAB) linker, VK (Val-Lys-PAB) linker, and hRS7-VK-PTX. **b** Reverse-phase (RP) HPLC analysis of the drug-to-antibody ratios (DARs) in different ADC molecules. hRS7:L0 + H0. ADC with DAR8:L1 + H3. **c** ADC molecules suppressed the growth of COLO205 cell-derived tumor xenografts. Two-tailed *t* test was used to assess statistical significance between treatment and control groups. ***P < 0.001, comparing hRS7-VK-PTX with hRS7; ^⊳⊳⊳^*P* < 0.001, comparing hRS7-VK-PTX with hRS7-VK-SN38 (*n* = 6 per group). Data = mean ± SD. **d** Dose-tolerability assay on hRS7-VK-PTX in BALB/c mice. **e** Treatment with both hRS7-VK-PTX and hRS7-VK-MMAE resulted in “bystander killing”, but hRS7-VK-PTX is more Trop-2-specific than hRS7-VK-MMAE (*n* = 5 per group). Data = mean ± SD. Right panel: schematic representation of “bystander killing” assay in mice. **f** Trafficking and cellular localization of anti-Trop-2 ADCs under confocal microscopy. Marker proteins LAMP-1, GM130, and clathrin (Red); anti-Trop-2 ADCs (Green); nuclear DNA (Blue). **g** ADC hRS7-VK-PTX internalized faster on MDA-MB-231 and CFPAC-1 cells analyzed by flow cytometry. **h** The suppression potency of free payload PTX and hRS7-VK-PTX on carcinoma cells with different Trop-2 expression levels

In cancer cell lines with diverse Trop-2 expression levels, our results indicated that free drug PTX is not as potent as MMAE, but similar to SN38 (Supplementary Table 1, Supplementary Figs. 3 and 4). As ADC, rather surprisingly, hRS7-VK-PTX was more potent than hRS7-VK-MMAE and hRS7-VK-SN38 in moderate Trop-2 expression cancer cell lines, including Capan-1, NCIH2452, MDA-MB-231, COLO205, and SK-MES-1 (Supplementary Table 2 and Supplementary Fig. 5). In addition, hRS7-VK-PTX even showed efficacy in PTX-resistant cancer cell lines, including SKBR3, CFPAC-1, MDA-MB-231, and COLO205 (Fig. 1h). Therefore, employment of a hydrophilic linker in PTX-conjugated ADC, as we hypothesized, significantly enhanced the anticarcinogenic potency of PTX in vitro.

The internalization rate of ADCs may impact their efficiency of cancer cell killing. Interestingly, internalization of hRS7-VK-PTX was faster than that of hRS7-VK-MMAE and hRS7-VK-SN38 (Fig. 1g). Under a confocal microscope, hRS7-VK-PTX molecules co-localized with lamp-1 and clathrin’s heavy chains, suggesting that they were internalized via clathrin-mediated endocytosis and trafficked into lysosomal compartments. In comparison, hRS7-VK-SN38 showed no obvious internalization (Fig. 1f). These results revealed that hRS7-VK-PTX can trigger a faster internalization rate, which may translate into superior therapeutic efficiency.

We then determined the therapeutic potentials of hRS7-VK-PTX in tumor xenograft models. hRS7-VK-PTX was more efficacious than hRS7-VK-SN38 in suppressing the growth of BXPC-3, COLO205, and HCC1806 cell-derived tumor xenografts (Fig. 1c; Supplementary Figs. 6 and 7). Moreover, hRS7-VK-PTX at 3 mg/kg (0.12 mg/kg PTX equivalent) was more efficacious than PTX at 10 mg/kg in suppressing the growth of BxPC-3 cell-derived xenografts (*P* = 0.0248, Supplementary Fig. 6a). In HCC1806 cell-derived models, the efficacy of hRS7-VK-PTX at 30 mg/kg (1.3 mg/kg PTX equivalent) was comparable with PTX at 10 mg/kg (Supplementary Fig. 6b). These results suggested that adoption of a hydrophilic linker in PTX-conjugated ADC significantly improved its antineoplastic effect in vivo.

We also verified the “bystander killing” of hRS7-VK-PTX in tumor xenograft models, which is necessary when targets are expressed heterogeneously in tumor tissues, and is driven by transferring of released payloads from the antigen-expressing cells to the neighboring antigen-absent cells. In our study, a mixture of Trop-2-positive BXPC-3 and Trop-2-negative NCIH1688 cells, or NCIH1688 cells alone were inoculated into BALB/c-nu/nu mice (Fig. 1e, right panel). The heterogeneity of Trop-2 expression in mixed cell-derived tumor xenografts and the negative control was confirmed by IHC (Supplementary Figs. 9b, c). Even though PTX as a free drug was much less potent than MMAE, treatment with both hRS7-VK-PTX (10 mg/kg) and hRS7-VK-MMAE (3 mg/kg) significantly suppressed co-inoculated tumor growth with comparable tumor growth inhibition (TGI) indexes at 99.5% and 91.7% (Fig. 1e; Supplementary Fig. 9a). However, hRS7-VK-MMAE, but not hRS7-VK-PTX, suppressed tumor growth in mice inoculated with NCIH1688 cells alone (Fig. 1e; Supplementary Fig. 9a), despite the absence of Trop-2 expression in tumor xenografts. These results confirmed that “bystander killing” from hRS7-VK-PTX exists and also suggested that the cell killing of hRS7-VK-PTX is more Trop-2-specific than that of hRS7-VK-MMAE, which may translate into a better safety profile clinically. Indeed, hRS7-VK-PTX (3 or 10 mg/kg) did not cause stress or body weight loss in treated mice (Supplementary Fig. 8), in sharp contrast to hRS7-VK-MMAE (3 mg/kg), hRS7-VK-SN38 (3 or 10 mg/kg), and PTX-alone treatment groups.

Last, we looked experimentally at the safety profile of hRS7-VK-PTX at relatively high doses in BALB/c mice. Although the maximum tolerable dose (MTD) of hRS7-VK-PTX was not reached due to limited reagent availability, it showed no obvious signs of toxicity or body weight loss at one single 100 mg/kg dosage when compared with placebo (Fig. 1d). In contrast, hRS7-VK-MMAE at only 60 mg/kg caused severe stress in mice as well as more than 15% of body weight loss (Supplementary Fig. 10). Therefore, hRS7-VK-PTX has a more favorable safety profile than that of hRS7-VK-MMAE.

To conclude, we have overcame the barrier of using PTX as ADC payload by introducing a hydrophilic linker. ADC molecules employing PTX, hydrophilic linkers, and Trop-2 antibodies showed superior efficacy and safety profile in vitro and in vivo, suggesting that it is a promising targeted therapeutic for human carcinomas.

## Supplementary information

Supplementary Information
