# Supplementary material for: Construction of paclitaxel-based antibody–drug conjugates with a PEGylated linker to achieve superior therapeutic index
Source: Signal Transduct Target Ther. 2020 Jul 29;5:132. doi: 10.1038/s41392-020-00247-y (PMC7391632; doi:10.1038/s41392-020-00247-y)
Supplement: Supplementary file 1 — Supplementary Information [file 41392_2020_247_MOESM1_ESM.docx]

**Supplementary Materials for**

**Construction of Paclitaxel-Based Antibody-Drug Conjugate with PEGylated Linker to Achieve Superior Therapeutic Index**

Ting Shao, Tianzhi Chen, Yuning Chen, Xiaoyue Liu, Yi-Li Chen,

Qi Wang, Tong Zhu, Maojun Guo, Hui Li, Dianwen Ju*, Chunhe Wang*

**^*^Corresponding to**: Chunhe Wang, 86-0760-88288712 phone; 86-21-50805906 FAX; [wangc@simm.ac.cn](mailto:wangc@simm.ac.cn), email or Dianwen Ju, 86-21-51980037 phone; [dianwenju@fudan.edu.cn,](mailto:dianwenju@fudan.edu.cn,) email.

**This file includes:**

Materials and Methods.

Supplementary Figure 1-10.

Supplementary Table 1-2.**Materials and Methods**

***Cell lines and reagents***

MDA-MB-468, BXPC-3, SKBR3, CFPAC-1, SCLC-21H, HCC1806, Capan-1, NCIH2452, MDA-MB-231, COLO205, HT29, NCI-H1688 and SK-MES-1 cells were purchased from Shanghai Cell Line Bank (Shanghai, China) and maintained in DMEM supplemented with 10% new born calf serum (Thermo Fisher Scientific, Waltham, MA, USA). The cells were incubated at 37℃ in humidified atmosphere of 5% CO2. PE-labeled goat anti-human IgG labeled was purchased from Biolegend (San Diego, USA). Humanized Trop-2 antibody hRS7 was prepared according to US patent 9931417 B2. Cell Counting Kit-8 (CCK8) was purchased from Dojindo Laboratories (Tokyo Japan). Matrigel (#354234) was purchased from BD Biosciences (San Jose, USA). Paclitaxel (PTX), MMAE and SN38 were synthesized by Levena Biopharma (Suzhou, China). Anti-LAMP-1 (#9091), anti-Clathrin (#4796), anti-GM130 (#12480) and Alexa Fluor® 555-anti-rabbit IgG F (ab') 2 Fragment were purchased from Cell Signaling Technology (Trask Lane Danvers, USA). IgG H&L (DyLight® 488) was purchased from Abcam Trading (Shanghai) Company Ltd. Human Trop-2 recombinant protein (6xHis Tag) was purchased from Sino Biological (Beijing China). Formaldehyde was purchased from Polysciences, Inc. (cat# 18814). Normal Goat Serum #5425 was purchased from Cell Signaling Technology (Trask Lane Danvers, USA). ProLong™Live Antifade Reagent (#P36974) and TMB substrate were purchased from Thermo Fisher Scientific Inc. TCEP (Bond Breaker™) was from Pierce (Rockford, IL, USA). Dimethylsulfoxide (DMSO) and other chemicals were products from [Sigma-Aldrich®](https://www.sigmaaldrich.com/catalog/search?interface=All_ZH&N=0+4294963318&mode=match+partialmax&focus=product&lang=zh&region=CN" \t "https://www.sigmaaldrich.com/_self" \o "Sigma-Aldrich<sup>®</sup>) (Merck KGaA).

***Expression and purification of hRS7***

The amino acid sequences of the light and heavy chains of hRS7 were from US patent 9931417 B2, and then reverse-transcribed into cDNA sequences by Vector NTI software. The DNA sequences was synthesized and subcloned into the pcDNA3.1 vector and amplified in E. coli bacteria. Purified plasmids were transfected into HEK293 cells by PEI. Cells were then cultured in suspension in [Gibco® FreeStyle™ 293 expression medium](http://www.so.com/link?m=aPpf3dqZVxF0EXaO368gR9ar1n+aWF9iF8UkeY0nEmaRwqe/Dgnsl8BTKImtym4bhu+vPbLQTnV/ACY1QQiIJk30LoiugUkQs8YAlSmR5YdobOpA8I/jKR8aRfCmm9d49SuBt4GZmevflZ5KoX5vlNw==). After 5 Days of culture, cell culture supernatant was collected and antibodies were purified by Protein A chromatography followed by Superdex 200 gel filtration.

***Synthesis of linker-payloads***

The synthesis of Mal-peg4-Val-Lys(peg24)-PAB-paclitaxel (VK-PTX), Mal-peg4-Val-Lys(peg24)-PAB-MMAE (VK-MMAE), Mal-peg4-Val-Lys(peg24)-PAB-SN38 (VK-SN38) were performed by Sorrento Therapeutics (San Diego, USA) as described in Patent CN108066772A.

***Antibody reduction and conjugation***

Antibody reduction was performed by adding 6 molar equivalents of TCEP (1mg/mL) into 8mg/mL of antibody solution contains 9% sucrose, 10mM acetate, 0.01% Tween-20, pH 5.0 at 37 ℃ for 2 hrs. After reduction, 20 molar equivalents of linker-payload per antibody sulfhydryl group was added and incubated at 4℃for more than 16hrs. Solution mixture was exchanged and concentrated by centrifugation (15 min at 4000rpm, Thermo Fisher ST40R TX-1) with Amicon Ultracontainer (50,000 MWCO, Millipore Corporation). The concentrations of ADCs were measured by a UV detector (Nanodrop 100, Thermo Fisher Scientific Inc.) at 280nm absorption. The DAR values of the final products were confirmed by reverse phase (RP) -HPLC and QTOF mass-spectrometer.

***RP-HPLC***

RP-HPLC analysis was performed on 1260 Infinity UHPLC (Agilent Technologies) on the following conditions: 1) Agilent PLRP-S 1000Å, 8μm, 4.6*250mm; 2) Mobile phase A：0.01% aqueous trifluoroacetic acid (TFA); 3) Mobile phase B: acetonitrile solution containing 0.01% TFA; 4) Gradient program: 0-3min (100%A), 3-25min (100%A-100%B), 25-30min (100%B); 5) Column temperature: 70℃; 6) Sample injection volume:15ul (1mg/mL). The degrees of hydrophobicity of ADC molecules are in proportion to the number of conjugated payloads and can be reflected by their retention times. Since the drug-linker has UV absorption, the average number of conjugated drug molecules per antibody molecule in ADC can be calculated according to the following expression: average number of conjugated drug molecules= (L0 peak area ratio×0+L1 peak area ratio×1+H0 peak area ratio×0+H1 peak area ratio×1+H2 peak area ratio×2+H3 peak area ratio×3)/100×2.

***SEC-HPLC***

SEC-HPLC method was employed to monitor aggregation and degradation of ADCs. After incubation at 60 ℃ for 1hr, samples were analyzed with a 1260 HPLC system (Agilent, Santa Clara, CA) on an Thermo MAbPac SEC-1, 5 µm, (7.8 × 300 mm) P/N 088460. Mobile phase: Phosphate buffered saline (PBS), 50 mM sodium phosphate containing 300 mM sodium chloride, pH 6.8. The column was maintained at 26℃ during the separation. The detector was set at 280 nm. Flow rate: 0.7 mL/min. Sample injection volume:15μl.

***SDS-PAGE***

Samples were boiled in 1× loading buffer for 10 min, samples containing 5µg of hRS7 or ADCs were resolved by SDS-PAGE (10%Bio-Rad Criterion Tris-HCl gels), then stained for 5 min with Coomassie blue, and imaged on BioRad ChemiDoc MP.

***Trop-2-expression level measurement***

Cells were incubated with 5μg/mL of hRS7 in ice-cold staining medium for 30 min on ice and washed twice with ice-cold staining media to remove unbound antibodies. Cells were then stained with PE-goat anti-human IgG (5μg/mL) in ice-cold staining medium for 30 min and washed twice. Cells were examined by flow cytometry.

***ELISA***

96-well Immuno-plates were coated with 2 µg/mL his-tagged antigen proteins (Sino Biological Inc.) overnight at 4℃. Plates were washed, blocked with 1% casein and then incubated with 10nM antibodies or ADCs for 1 hr at room temperature. After washing, anti-human kappa-HRP (Biolegend San Diego, USA) was added and incubated for 1 hr. After washing, TMB substrate was added and detected at 450 nm with a SpectraMax M5e (Molecular Devices) microplate reader.

***Internalization assay***

To evaluate the internalization rate of ADCs, MDA-MB-231 or CFPAC-1 cells (1×10^6^) were incubated with 5μg/mL ADC samples in staining media for 30 min on ice and washed twice, before placed in fresh complete media at 37℃ humidified chamber to initialize internalization. At the end of incubation, cells were washed twice with ice-cold staining medium and then stained with goat anti-human IgG-PE. The cells were analyzed by flow cytometry to detect the level of remaining surface ADCs.

***Confocal microscopy***

SKBR3 cells were incubated with 5μg/mL hRS7-VK-PTX or hRS7-VK-SN38in 24-well plates (1×10^4^ cells/ well) for 45 or 90min at 37℃ in 5% CO_2_. The cells were washed once in PBS, fixed and permeabilized. After washing, the cells were stained with DyLight®488-goat anti-human IgG (H&L) or Alexa Fluor® 555-anti-rabbit IgG F(ab') 2 Fragment. Nuclear DNAs were stained with 4',6-diamidino-2-phenylindole (DAPI). Immunofluorescence was recorded with an Olympus BK71 confocal microscope.

***Cell viability assays***

Cell viability was determined using Cell Counting Kit-8 (CCK-8). Cells were seeded into 96-well plate at 3000-10000 cells/well. After incubation overnight，drugs were added to different concentrations. Plates were incubated in a humidified chamber for 96 h at 37℃，5% CO_2_. CCK8 agent was added and placed back into the incubator until untreated control cells had an absorbance greater than 1.0 at 450nm. Growth inhibition was measured as percentage of growth relative to untreated cells. Dose-response curves were generated from the mean of twice or triplicate determinations, and IC_50_values were calculated using Prism GraphPad Software.

***Mouse xenograft models***

Five-week-old female athymic BALB/c nu/nu mice were purchased from Charles River Company (Shanghai, China) and randomly divided into groups of 5-6 mice each. Animal handling and procedures were approved and performed according to the requirements of the Institutional Animal Care and Use Committee (IACUC) of Shanghai Institute of Materia Medica, CAS. All models were established by s.c. inoculation in the flanks of the mice. BXPC-3, HCC1806, and NCIH1688 cells were established by injecting 5×10^6^ cells suspended in a Matrigel matrix. COLO205 cells were established by injecting 1×10^7^ cells suspended in a Matrigel matrix. After tumor volume reached about 200mm^3^, tumor-bearing mice were randomized into treatment and control groups based on tumor volumes. Each drug was administered i.p. to the mice at 3 or 10 mg/kg (10 mL/kg). The tumor volume was defined as length×1/2× width×width. The percentages of inhibition were calculated by formula: (1-average tumor volume of treatment group/average tumor volume of control group)×100%. Toxicity associated with different treatment group was evaluated by monitoring the body weight loss.

***Dose tolerability assay***

Dose tolerability assay was performed in BALB/cmice (3 mice per dose). Drugs were administered as a single i.v. injection at 60, 80 and 100 mg/kg for hRS7-VK-PTX and 60 and 80mg/kg for hRS7-VK-MMAE. Toxicity was assessed by observing mouse behavior, weight loss, and survival. The percentages of body weight change (%) was calculated as: (body weight-body weight before treatment) / (body weight before treatment) ×100%.

***Statistical analysis***

Prism GraphPad version 6 was used for statistical analysis. Results are shown as mean ± SD. Statistical differences between control and experimental groups were calculated by Student’s t-test, with P < 0.05 considered significant.

**Supplementary Figures:**

**
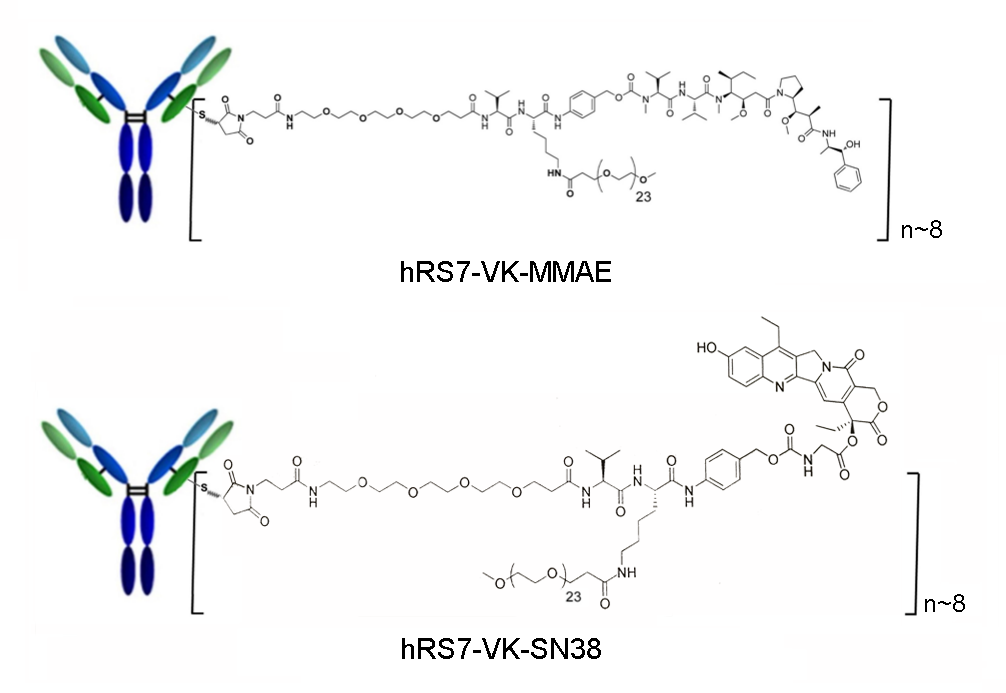
**

**Supplementary Figure 1:** Molecular structures of hRS7-VK-MMAE and hRS7-VK-SN38.


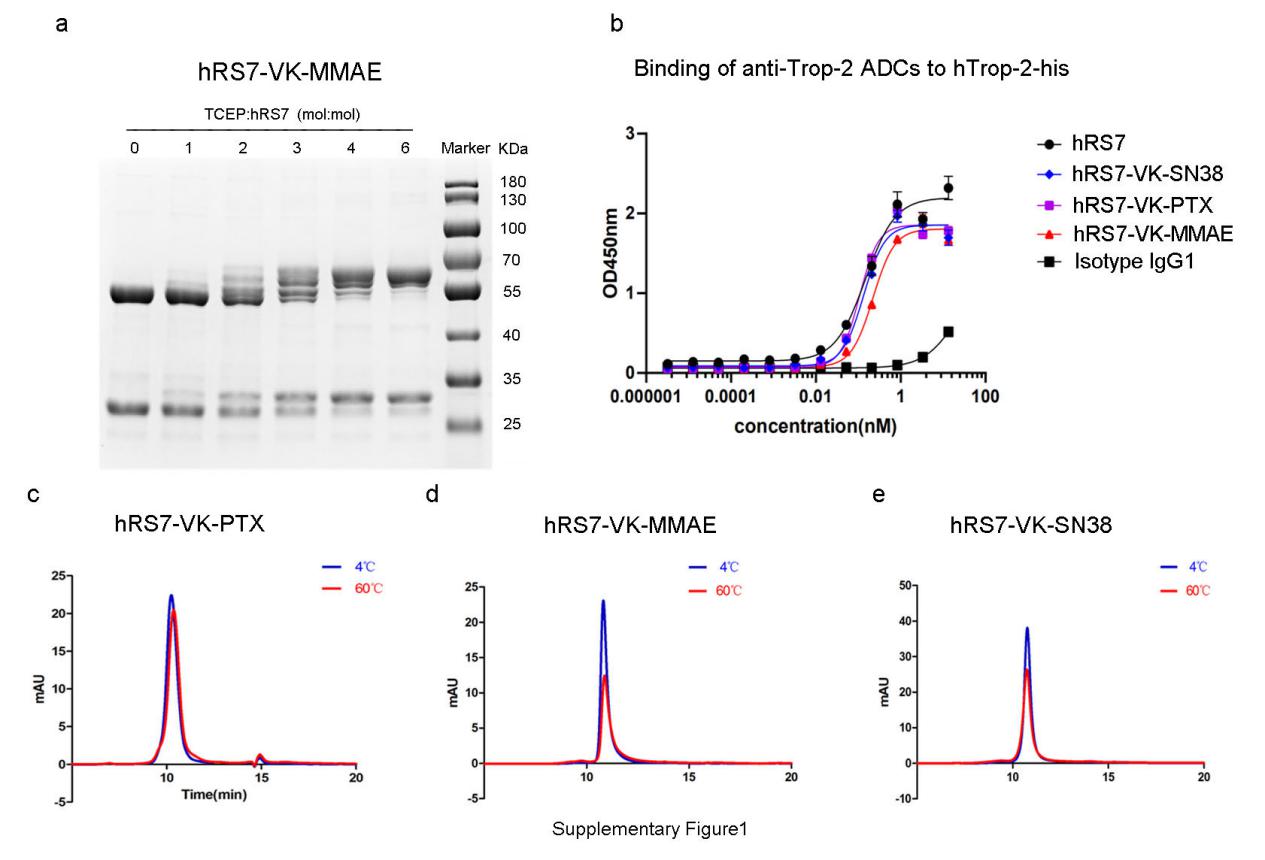


**Supplementary Figure 2:** Biochemical characterization of anti-Trop-2 ADCs. **a**, SDS-PAGE analysis of hRS7-VK-MMAE with DAR values from 0 to 8 under reducing condition. **b,** Affinity of different ADC molecules with DAR value at 8 to human Trop-2protein (6xHis tagged). Data=mean± SD. **c,d and e**, ADC molecules with different payload at DAR = 8 showed no significant aggregation and degradation on SEC-HPLC after incubation at 60℃ or 4℃ for 1 hr. After incubation at 60 ℃ for 1hr.

**
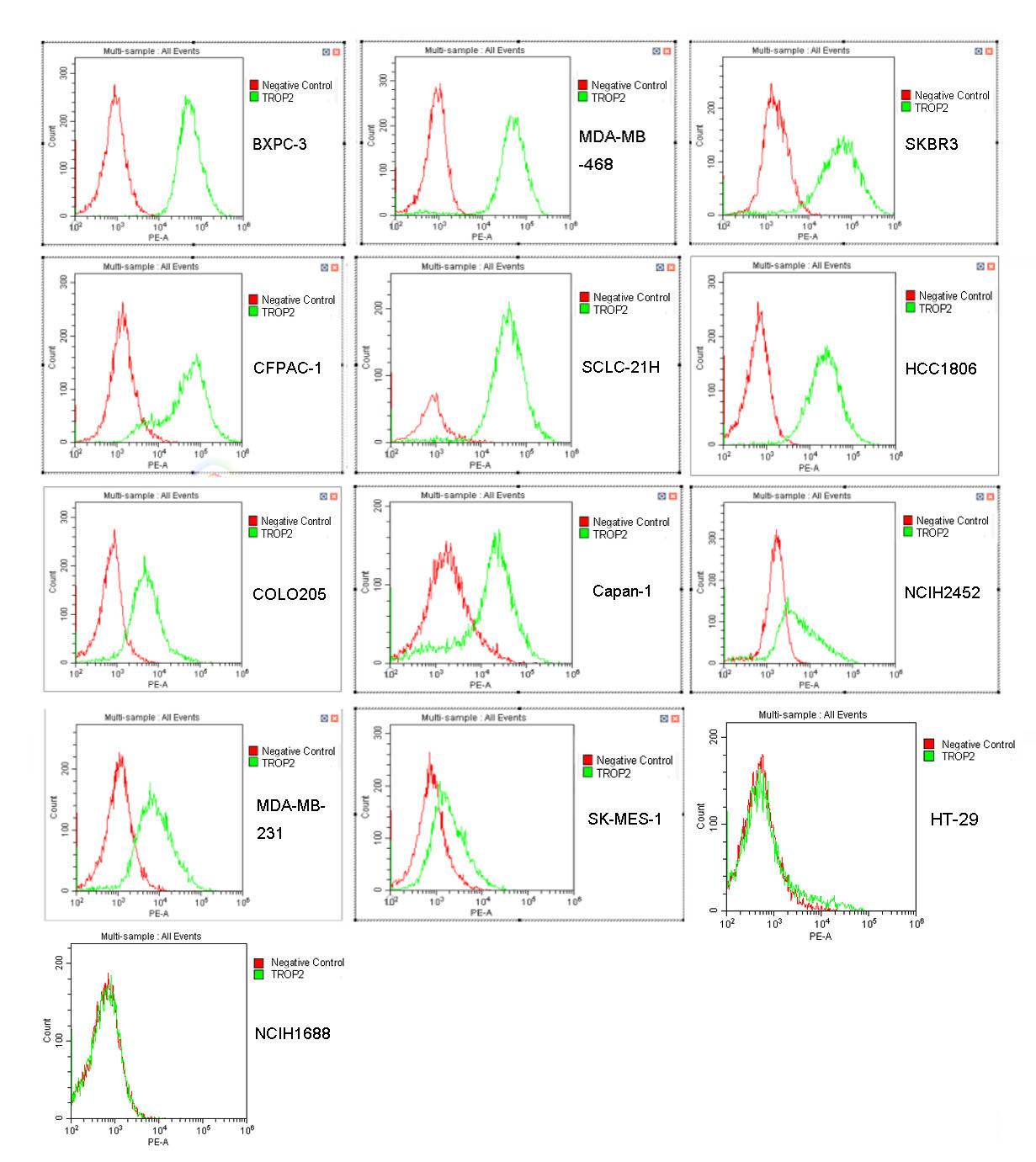
**

**Supplementary Figure 3.** Analysis of Trop-2 expression levels on different cancer cell lines by flow cytometry. Cancer cells (1×10^6^ cells/mL) in PBS were incubated at 4℃ with 5μg/mL hRS7 for 30 minutes. Human IgG1 was used as isotype control.

**
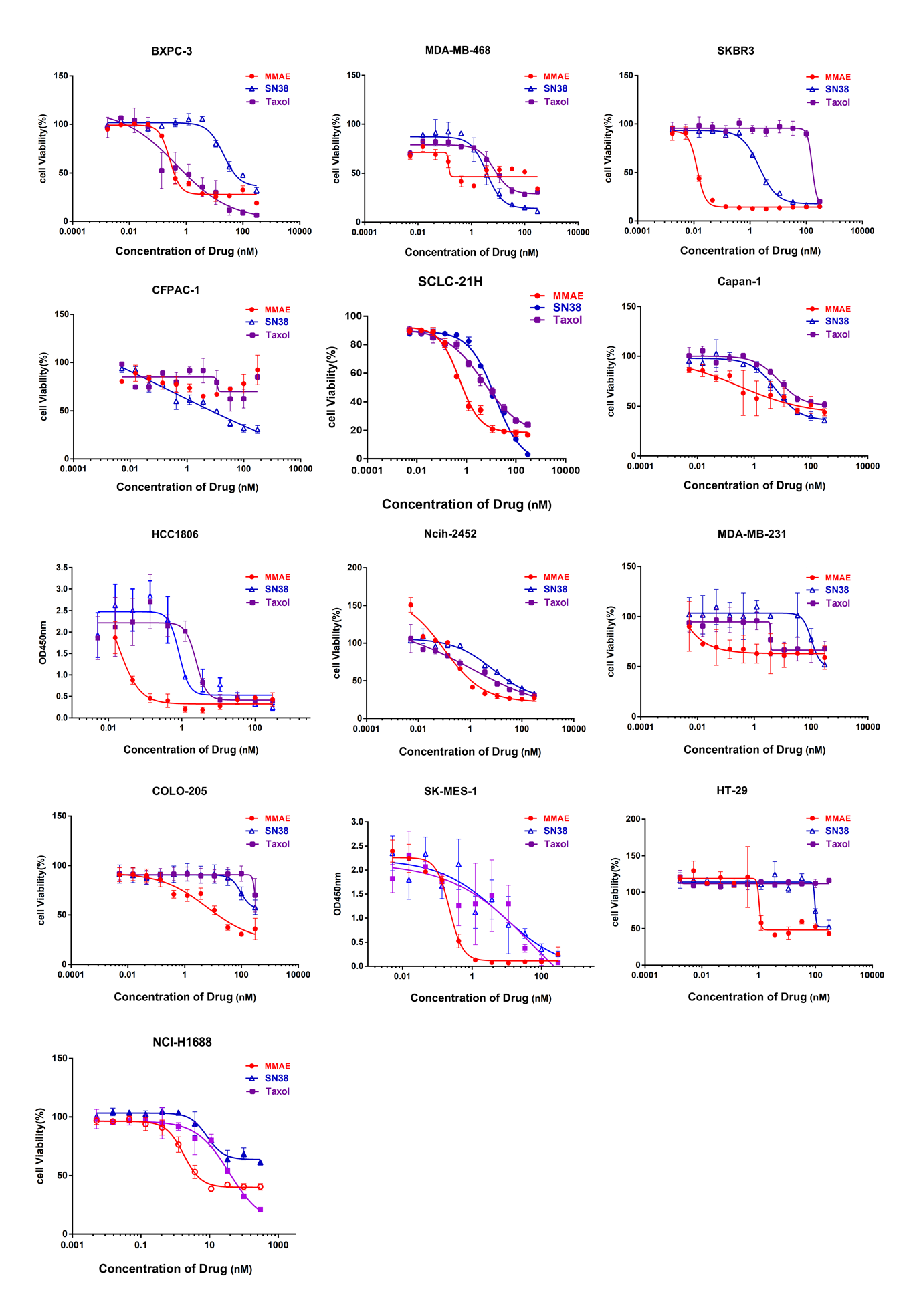
**

**Supplementary Figure 4.** The anti-neoplastic efficacies of different free payloads in different carcinoma cell lines assessed by CCK-8 assay after 96 h of treatment. Data = mean ± SD.

**
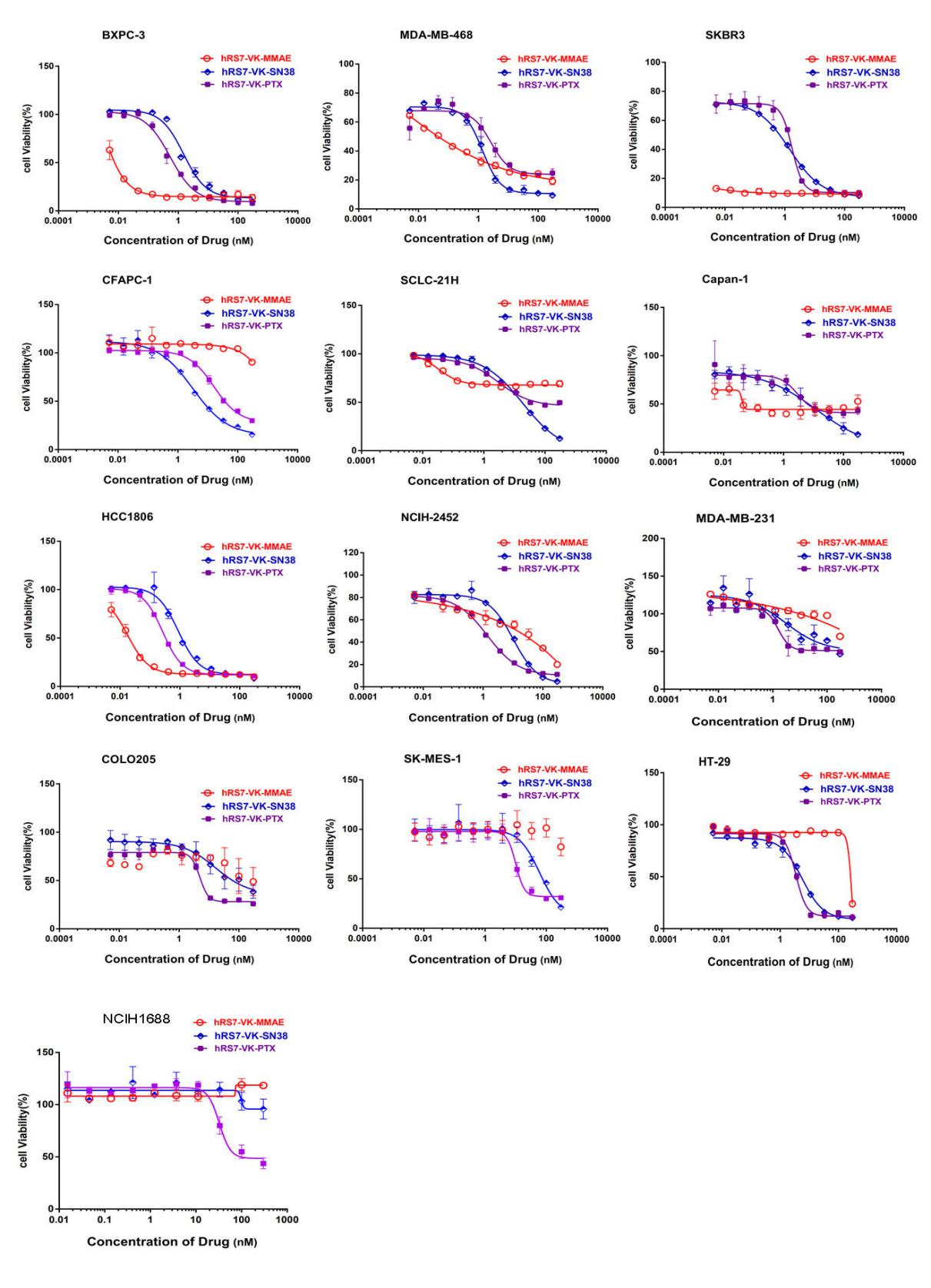
**

**Supplementary Figure 5.** The anti-neoplastic efficacies of ADC molecules in different carcinoma cell lines. Cytotoxicity of ADCs in various Trop-2-expressing cancer cell lines were assessed by using the Cell Counting Kit-8 assay after 96 h of treatment. Data = mean ± SD.

**
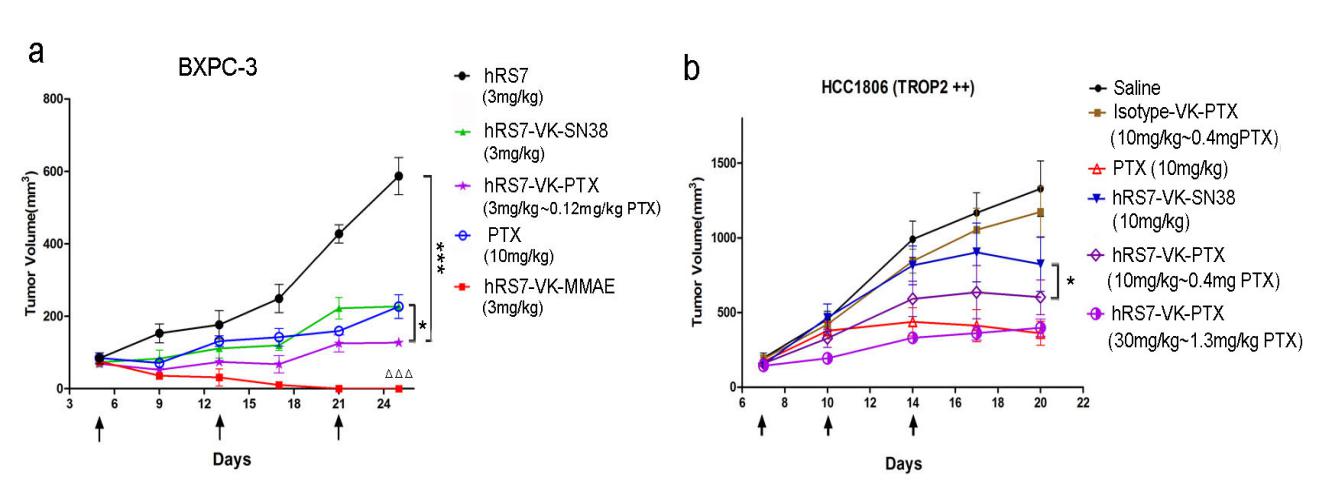
**

**Supplementary Figure 6.** ADC molecules suppressed tumor growth in mouse xenograft models. **a,** Mice inoculated with BXPC-3 cells (n = 5 per group) were treated with ADCs or naked antibody on Days 5, 13 and 21. Tumor were measured twice or thrice per week. Two-tailed t-test was used to assess statistical significance between treatment and control groups on Day 25. ***P < 0.001, comparing to hRS7 treatment group; *P < 0.05, comparing hRS7-VK-PTX to PTX treatment group; ^▷▷▷^P<0.001, comparing to hRS7 treatment group. Data = mean ± SD. **b,** Mice inoculated with HCC1806cells (n=6 per group) were treated on Days 7, 10 and 14. Tumor were measured twice or thrice per week. Two-tailed t-test was used to assess statistical significance between treatment and control groups on Day 20. *P < 0.05 comparing hRS7-VK-PTX to hRS7-VK-SN38 treatment groups. Data = mean ± SD.

**
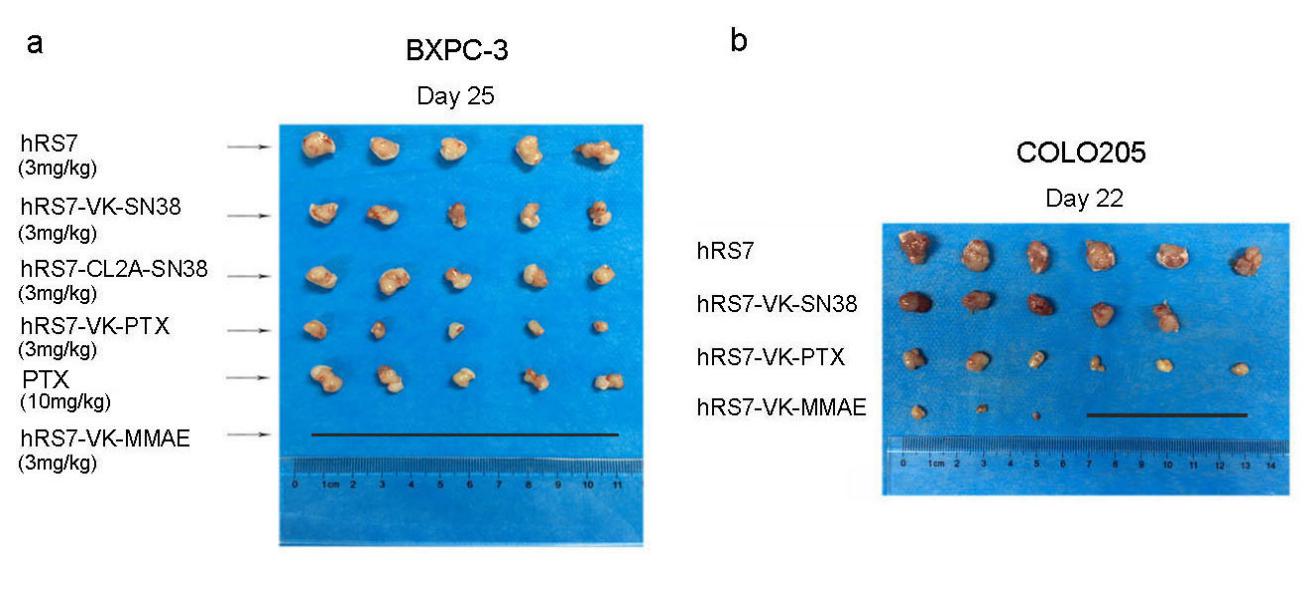
**

**Supplementary Figure 7**: Comparison of tumor tissues dissected from mouse xenograft models treated with different ADC molecules. ADC treatment suppressed tumor growth in BXPC3 (**a**) or COLO205 cell-derivated (**b**) mouse xenograft models. Tumor tissues were collected on Day 25 (**a**) or Day 22 (**b**)."▁"[represent](javascript:;)s complete suppression.


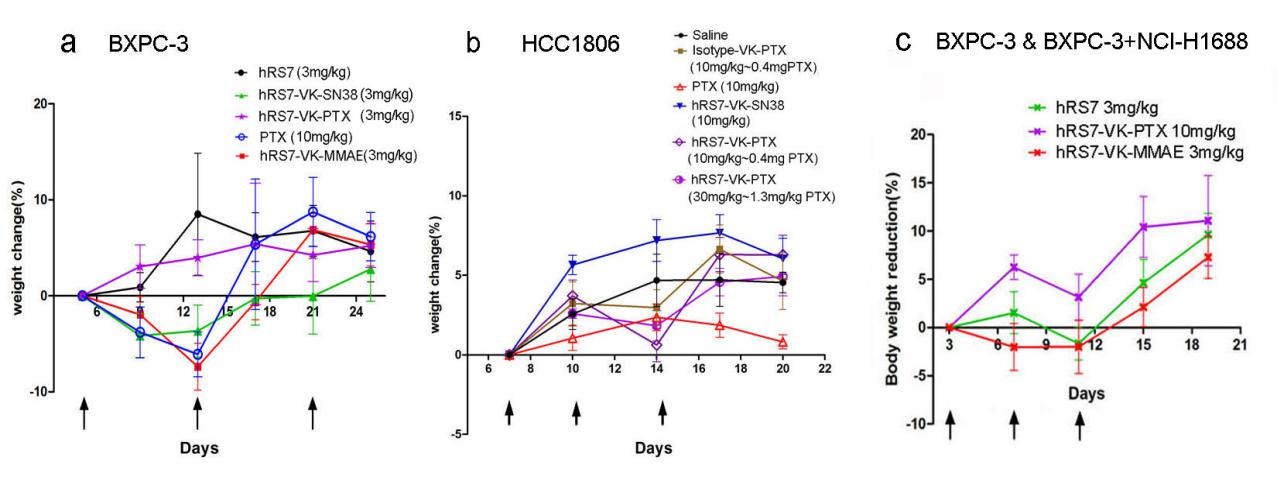


**Supplementary Figure 8.** Body weight change of ADC-treated mice. Data = mean ± SD.


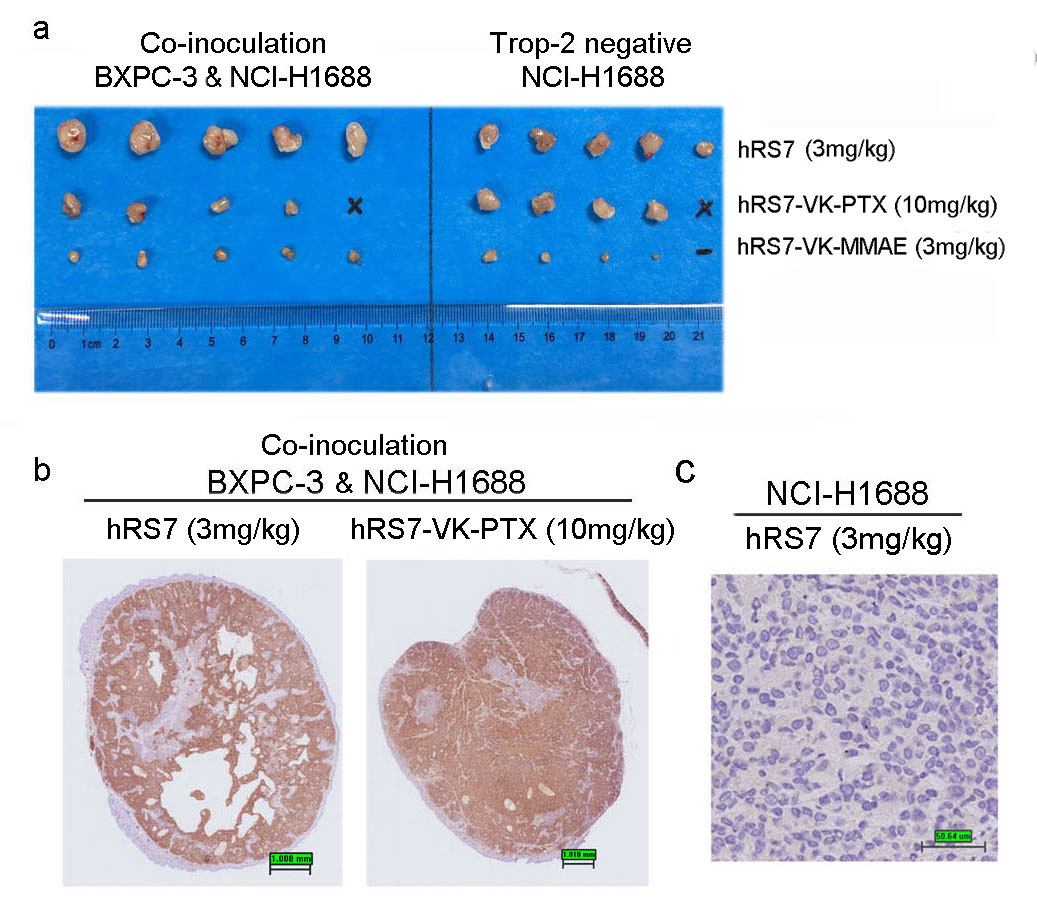


**Supplementary Figure 9**: Anti-neoplastic activity of Trop-2 ADCs and their “bystander killing”. **a**, Mice bearing BXPC-3 and NCIH1688 cell mixture-derived tumors, or NCIH1688 cells alone-derived tumors (n = 5 per group) were treated with hRS7-VK-PTX (10 mg/kg) and hRS7-VK-MMAE (3 mg/kg) on Days 3, 7 and 11. Tumor volumes were measured twice or thrice per week and shown in Fig.1e. Tumor tissues were collected on Day 19."▁"[represent](javascript:;)s tumors complete inhibited. "×" represents absence due to death of mice **b,** Trop-2-expression in co-inoculated tumors as revealed by IHC. 19 Days after s.c. inoculation, tumors were collected, fixed and stained for Trop-2 by IHC. Bar = 1mm. **c.** Trop-2 expression is absent in NCI-H1688 cells-derived tumors as confirmed by IHC. 19 Days after s.c. inoculation, tumor tissues were collected, fixed and stained for Trop-2 by IHC. Bar = 50 µm.


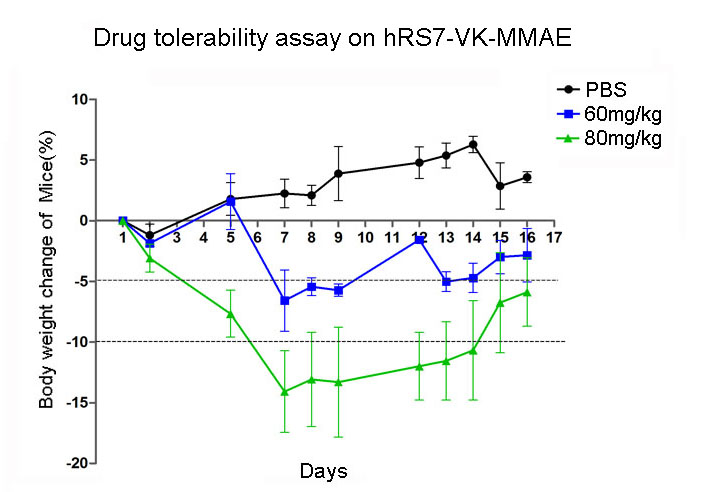


**Supplementary Figure 10:** Drug tolerability assay on hRS7-VK-MMAE. MTD was determined in BALB/c mice (3 mice per dose) by a single tail vein injection of hRS7-VK-MMAE or placebo. Mice were observed for 16 Days and toxicity was assessed by observing mice’ behaviors, weight losses, and survival rates. Data = mean ± SD.


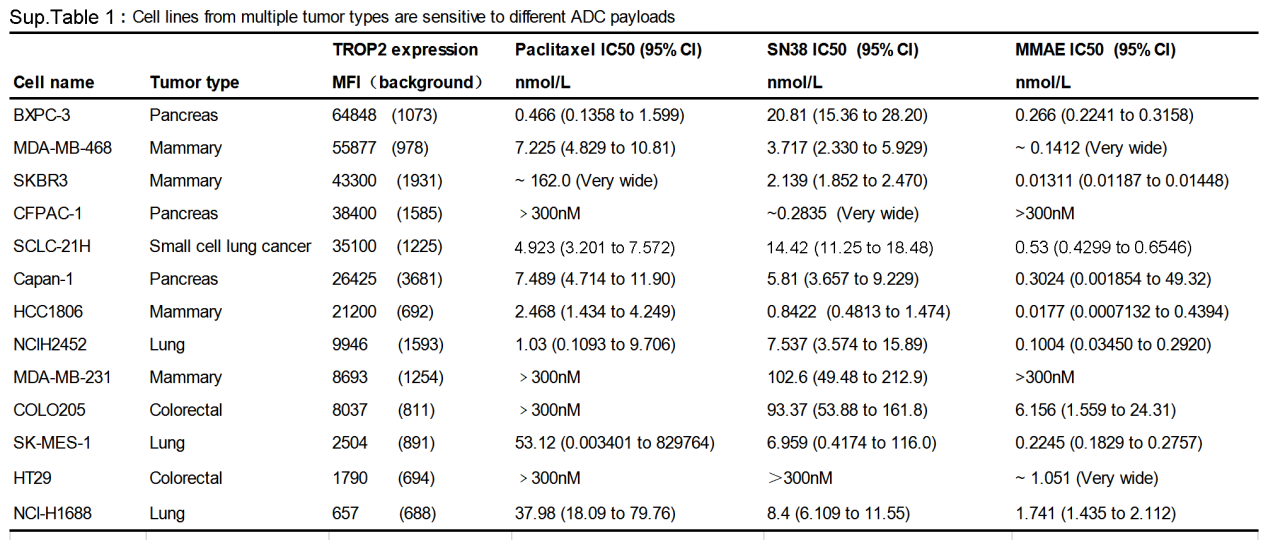


**Supplementary Table 1:** The suppression potency of free payload PTX, MMAE and SN38 on carcinoma cells with different Trop-2 expression levels.


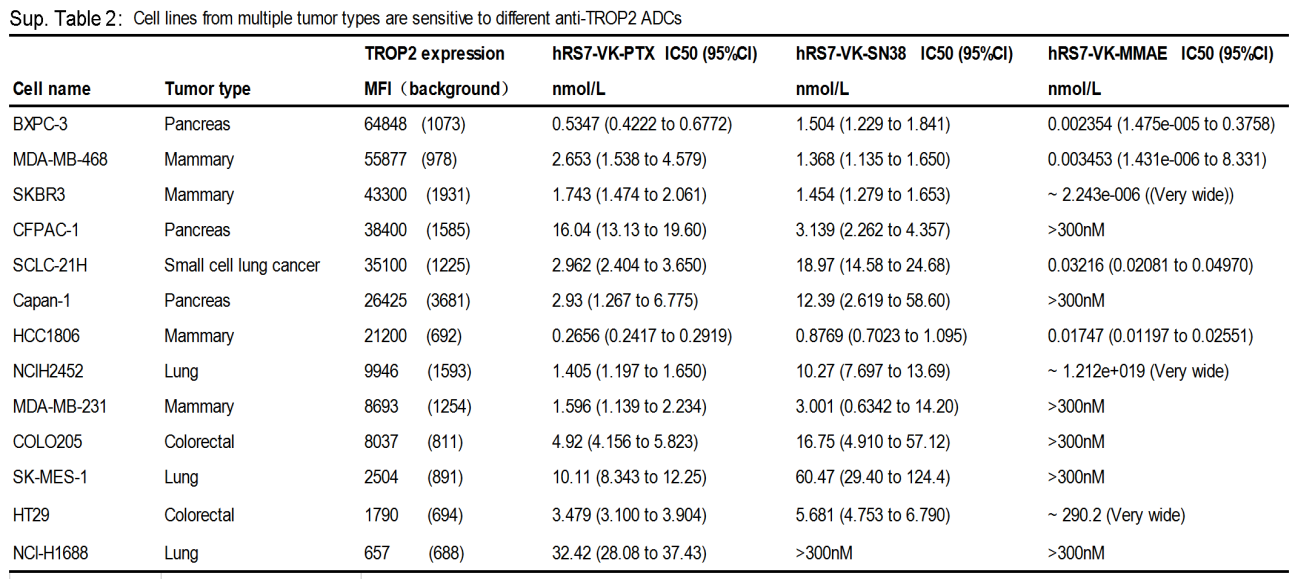


**Supplementary Table 2:** Compare the potency of hRS7-VK-PTX, hRS7-VK-MMAE and hRS7-VK-SN38 in different carcinoma cell lines with diverse Trop-2 expression levels.
